# Effects of dietary Trollius chinensis Bunge residue supplementation on growth performance, antioxidant status, intestinal morphology, and cecal microbiota in weaned rabbits

**DOI:** 10.3389/fvets.2025.1640419

**Published:** 2025-09-19

**Authors:** Lingcong Deng, Juan Fang, Jiayu Yu, Yu Dong, Kailong Han, Xue Yang, Dongliang Fei, Xibin Han, Mingxiao Ma, Jieying Bai

**Affiliations:** ^1^Collaborative Innovation Center for Zoonosis Prevention and Control, Jinzhou Medical University, Jinzhou, China; ^2^College of Veterinary Medicine, Nanjing Agricultural University, Nanjing, China; ^3^Xiangtan Animal Disease Prevention and Control Center, Xiangtan, China; ^4^College of Veterinary Medicine, Yangzhou University, Yangzhou, China; ^5^College of Animal Science and Veterinary Medicine, Shenyang, China; ^6^Jilin Genet-Med Biotechnological Co., Ltd., Changchun, China; ^7^Genet-Med (Dezhou) Biotechnological Co., Ltd., Dezhou, China

**Keywords:** traditional Chinese medicine residues, Trollius chinensis Bunge, rabbit, cecal microbiota, antioxidant capacity, blood biochemistry

## Abstract

**Background and objective:**

Weaning stress can cause decreased immunity and intestinal flora imbalance, leading to diarrhea and even death of the rabbits. The present study aimed to investigate the benefits from Trollius chinensis Bunge residues (TCBR) on growth performance, antioxidant capacity, intestinal health and cecal microbiota in weaned rabbits.

**Methods:**

Through the ultra performance liquid chromatography (UPLC) technology, the main active ingredients from TCBR were analyzed. And then, 48 30-day-old rabbits were randomly allocated into 4 groups, with 12 replicates per group. Four diets were formulated with graded levels of TCBR: 2.0, 4.0, and 6.0% represented as TCBR2, TCBR4, and TCBR6 groups alongside a Mock group without TCBR.

**Results:**

Our results showed that TCBR2 significantly alleviated adverse clinical manifestations in weaned rabbits and improved survival rate, growth performance, and reduced the feed conversion ratio compared with the Mock group. TCBR2 also enhanced carcass yield, partial-eviscerated carcass yield, and antioxidant capacity, and increased jejunal villus height and villus/crypt ratio compared with that in the Mock group, whereas no differences were observed between the TCBR4 and TCBR6 groups. Furthermore, TCBR2 significantly increased the expression levels of Occludin and ZO-1 in jejunal tissue while reducing the expression levels of TNF-*α* and IL-8. Notably, 16S RNA analysis revealed that *Bacteroidota* levels were significantly elevated in the TCBR2 groups, with *Akkermansia*, *Clostridium*, and *Succiniclasticum* also up-regulated in the TCBR2 group.

**Conclusion:**

TCBR2 supplementation improved growth performance and attenuated adverse clinical symptoms in rabbits, suggesting the potential of low-dose TCBR as a feed additive.

## 1 Introduction

Rabbit meat is highly nutritious and healthy, containing high levels of polyunsaturated fatty acids, proteins, and essential amino acids, which benefit the human diet (1). Rabbit meat production primarily occurs in Asia, Europe, the Americas, and Africa, with Asia accounting for 70% of global production. China leads the Asian rabbit meat market (2). The first 10–15 days after weaning are the most critical period of postnatal development for rabbits. During this period, weaned rabbits are susceptible to gastrointestinal infections, leading not only to increased mortality but also to growth retardation and consequently severe economic losses (3). Especially, the implementation of the “Prohibition of Antibiotic Use,” policy has worsened health issues in Chinese rabbit farms (4, 5). Therefore, nutritional regulation using exogenous supplements to promote the healthy development of the livestock and poultry industry, has become a research focus (6–9).

Traditional Chinese medicine (TCM), natural substances known for their safety, simple preparation, affordability, and minimal side effects (10, 11), has been widely used to prevent and treat various diseases in humans and animals, including as herbal antimicrobial, anti-inflammatory, antiparasitic, and antidiarrheal agents (12–15). Several studies have suggested that herbal ingredients in animal feed can promote growth, intestinal integrity, antioxidant effects, nutrient absorption, and immunity (16–20). Researchers demonstrated that residues of TCM are abundant in bioactive compounds, offering numerous nutritional and health benefits to animals. Animal health is intricately linked to immune homeostasis, with the removal and suppression of inflammatory factors being crucial for maintaining health. The NF-κB pathway is a critical regulator of the expression of various pro-inflammatory factors and serves as a key mediator of the inflammatory response. Several studies indicated that TCM residues modulated the expression of inflammatory factors via the NF-κB pathway, thereby exerting a beneficial effect on animal physiology (21–23). Furthermore, some TCM residues mixture showed to reduce the expression levels of inflammatory markers such as IL-4, IL-1β, and TNF-*α*, which in turn enhances the egg production of chickens (24). Additionally, the intestinal tract, the body’s largest immune organ, plays a significant role in immune modulation through its resident intestinal flora. Dietary supplementation with Isatidis root residue showed to reduce the prevalence of detrimental intestinal bacteria, such as *Campylobacter*, *Actinobacillus minor*, and *Ralstonia pickettii*. These reduction contributes to the enhancement of intestinal barrier integrity and mitigates diarrhea associated with early weaning (25). Furthermore, Sun et al. demonstrated that Xiasangju residue increased the relative abundance of beneficial bacteria, including *Lactobacillus johnsonii* and *Weissella jogaeotgali*, while decreasing the relative abundance of harmful bacteria such as *Escherichia coli* and *Treponema porcinum*. Additionally, this supplementation led to an increased expression of IL-10 and a decreased expression of IL-1β in the ileum, thereby improving the integrity of the intestinal tight junction barrier (26). *Trollius chinensis Bunge*, a perennial herb of the buttercup family, has high medicinal value owing to its anti-inflammatory, antioxidant, antibacterial, and antiviral properties. These benefits are attributed to its rich bioactive compounds, such as flavonoids, organic acids, and alkaloids, evaluated the toxicity of *T. chinensis*, confirming its low toxicity and safety in animal feed (27–29). However, To date, no studies have evaluated the effects of *Trollius chinensis Bunge* residues (TCBR) in meat rabbit production.

This study aimed to investigate the effects of TCBR supplementation on the growth performance, serum biochemical indexes, antioxidant capacity, and cecal microbiota of weaned rabbits, providing a theoretical basis for using TCBR as a feed additive to promote healthy rabbit breeding.

## 2 Materials and methods

### 2.1 Trollius chinensis Bunge residues composition assay

The dried residues of *Trollius chinensis Bunge* were pulverized and placed into a medicinal bag for a 2-h soak. Subsequently, the contents were brought to a boil and decocted twice, followed by concentration to obtain the aqueous extract of *Trollius chinensis Bunge* residues. A 200 μL aliquot of the drug solution was mixed with 800 μL of methanol, subjected to vortex mixing for 10 min, and then centrifuged at 13,000 rpm for 10 min. The supernatant was collected for analysis. Chromatographic separation was performed using a Welch AQ-C18 column with a mobile phase comprising 0.1% formic acid in water (solvent A) and methanol (solvent B). The elution was executed under the following gradient conditions: 0–10 min, 2–20% B; 10–15 min, 50–80% B; 15–20 min, 80–95% B; 20–27 min, 95% B; 27–28 min, 95–2% B; and 28–30 min, 2% B. The injection volume was 5 μL, with a flow rate of 0.3 mL/min, and the detection wavelength was set at 254 nm. Additionally, a Q Exactive high-resolution mass spectrometer was employed for analysis, utilizing an electrospray ionization (ESI) source and operating in both positive and negative ion modes. Furthermore, a Q Exactive high-resolution mass spectrometer was employed for the analytical procedures. This instrument utilizes an electrospray ionization (ESI) source and is capable of operating in both positive and negative ionization modes. Data acquisition was conducted using data-dependent acquisition (DDA), with dynamic background subtraction (DBS) performed at 30-s intervals, and a high sensitivity mode set at 70,000 resolution. Preliminary organization of the high-resolution liquid chromatography data was facilitated by Compound Discoverer 3.3 (CD 3.3, Thermo Fisher), followed by database searching against mzCloud.

### 2.2 Experimental design, diets, growth performance, and clinical records

In total, 48 weaned (30-days-old) weaned rabbits were used in a 42-day experiment. Each rabbit was housed individually, with each cage representing a replicate experimental unit. After assessing their initial body weight (BW), the weaned rabbits were randomly assigned to one of four dietary groups (*n* = 12/group), with TCBR added to their diets as follows: (1) Mock without TCBR, (2) TCBR2 containing 2% TCBR, (3) TCBR4 containing 4% TCBR, and (4) TCBR6 containing 6% TCBR. The basal diet, containing corn and soybean meal, was formulated to meet or exceed the nutrient requirements for growing rabbits. The ingredients and proximate composition of the basic diets are provided in Table 1. Rabbits had free access to feed and water without any vaccinations during the experiment. Individual feed intake was recorded daily, and BW was recorded once every 5 days daily feed intake, body weight gain, and feed conversion ratio (FCR = feed intake/body weight gain) were calculated. Clinical scores were based on the Vesikari rating system with slight modifications (30). A five-point scale from 1 to 5 was used, reflecting normality (0 score), non-feeding (1 score), mild diarrhea (2 score), severe diarrhea (3 score), and death (5 score).

**Table 1:** Composition and nutrient content of the diet for rabbits aged 30–72 days (as-fed basis).

Item	Value
Ingredients	
Corn, %	28.0
Soybean meal, %	12.0
NaCl, %	0.3
Trace element, %	0.5
CaHPO_4_.2H_2_O, %	1.8
Multi-vatimins, %	0.05
Choline chloride, %	0.2
lucerne, %	36.0
Semipowder, %	17.0
Rice brane, %	3.2
Vegetable oil, %	0.5
Talcum powder, %	0.5
Total, %	100.5
Nutrition contents^1^	
Moisture, g/kg	72.0
Crude protein, g/kg	170.5
Crude fat, g/kg	30.0
Crude fiber, g/kg	142.0
Crude ash, g/kg	81.0
Calcium, g/kg	10.4
Total phosphorus, g/kg	5.6

1 Values were derived from the Chinese nutrient requirements of rabbits.

### 2.3 Sample collection and calculation of slaughter performance

After 42 days of feeding, the rabbits were fasted overnight before sampling and slaughter. The 8 rabbits were randomly selected from 12 rabbits per group and sacrificed via intravenous air injection at the ear margin. Blood samples were collected via heart puncture, centrifuged to collect serum, and stored at −20°C for further analysis. Eight rabbits from each group were selected to assess slaughter performance. The rabbits were sequentially skinned, limbed, decapitated, and eviscerated. The following measurements were recorded: carcass weight, semi-eviscerated carcass weight, and eviscerated carcass weight. Carcass traits were calculated relative to live BW based on previous studies and slightly revision (31).


Carcass yield(%)=Slaughter weight/live weight×100%.

$$\begin{array}{l} \text{Partial eviscerated weight yield}\;(\%)=\text{partial eviscerated weight}\\ /\text{live weight}\times 100\%. \end{array}$$

$$\begin{array}{l} \text{Eviscerated carcass yield}\;(\%)=\text{Eviscerated carcass weight}\\ /\text{live weight}\times 100\%. \end{array}$$

### 2.4 Measurement of serum biochemistry

Serum levels of glucose (GLU), triglyceride (TG), total cholesterol (T-CHO), albumin (ALB), alanine aminotransferase (ALT), blood urea nitrogen (BUN), alkaline phosphatase (ALP), and total protein (TP) were assayed using ELISA kits (F006-1-1, A110-1-1, A111-1-1, A028-2-1, C009-2-1, C013-2-1, A059-2-2, and A045-2-2; Nanjing Jiancheng Institute of Bioengineering, Nanjing, PRC) and a microplate reader (Tecan Austria GmbH, Vienna, Austrian).

### 2.5 Determination of antioxidant parameters

Serum and liver antioxidant parameters were measured using a previously described method (6). Briefly, serum samples were directly analyzed, whereas liver samples were homogenized with precooled phosphate-buffered saline (1:10, v/v) and centrifuged (8,000 rpm and 4°C for 10 min) to obtain clarified homogenates. The activities of total superoxide dismutase (SOD), catalase (CAT), and glutathione peroxidase (GSH-Px), along with the concentration of malondialdehyde (MDA), were assayed using ELISA kits (A001-3-2, A007-1-1, A005-1-2, and A003-1-1; Nanjing Jiancheng Institute of Bioengineering) and a spectrophotometer, as described above.

### 2.6 Morphological observation of the ileum wall

Fixed ileum samples were dehydrated, embedded in overheated paraffin, and cut into 4-μm-thick sections (RM2235, Leica Microsystems, Germany). After drying, dewaxing, and staining with hematoxylin and eosin, the sections were scanned using a Pannoramic Scanner (C13210-01, HAMAMATSU, Japan). Villus height and crypt depth were measured using ImageJ software (version 1.54), and the villus-to-crypt (V/C) ratio was calculated.

### 2.7 RNA extraction, cDNA synthesis, and quantitative real-time PCR

Tissue RNA was extracted according to methods described in a previous study (32). Ileum tissues were homogenized with precooled phosphate buffered saline (PBS), and the homogenates were lysed using RNA isolator total RNA extraction reagent (Vazyme Biotech, Nanjing, China). RNA concentration was measured using a NanoDrop 2000 spectrophotometer (Thermo Fisher Scientific, MA, USA). Subsequently, a cDNA template was synthesized from 1,000 ng of RNA using the HiScript®II Q RT SuperMix for quantitative PCR (qPCR; +gDNA wiper; Vazyme Biotech). Real-time qPCR was performed on an ABI system (Thermo Fisher Scientific) using the ChamQTM SYBR® qPCR Master Mix (Vazyme Biotech). PCR conditions comprised 40 cycles of 95°C for 5 s, 60°C for 30 s, and 72°C for 60 s. The sequences of the primers used are listed in Table 2. Gene relative expression was calculated using the 2^−∆∆Ct^ method.

**Table 2:** Primers used for gene expression analysis via qPCR.

Gene	Primer sequence (5′-3′)	Accession No.	Product size (bp)
TNF-α	F: AAGGTCAACCTCCTCTCT	NM_001082263	93
	R: CAGGTAGATGGGCTCGTACC		
IL-8	F: TGATGGAAGAGAACTCTGC	NM_001082293	92
	R: ATGACTCTTGCTGCTCAG		
IL-1β	F: GAATTTGAGTCTGCCCAGTT	NM_001082201	104
	R: CCATGCTGAAGTCAATTAGGT		
ZO-1	F: GAGAACAAGAAGGAGGTGAA	NM_100346390	317
	R: CACTGAACTGGCTCTGAG		
Occludin	F: TTGAGCAGCAGCAGTAAC	NM_100338492	427
	R: TGTAGTCCGTCTCGTAGTG		
Claudin-1	F: AATTCGGTCAGGCTCTTT	NM_001089316	96
	R: GAGGACAAGAACAGCAAAG		
GAPDH	F: TTCCCGTTCTCAGCCTTGACC	NM_001082253.1	118
	R: TGCTGATGAGTACAACCGACT		

### 2.8 Analysis of the cecal microbial community

Total DNA from cecal samples was extracted using the Cetyltrimethylammonium Bromide (CTAB) method according to the manufacturer’s instructions. Sequences of the 16S rRNA genes from the V3–V4 hypervariable regions were amplified using the universal primers 341F (5′-CCTACGGGNGGCWGCAG-3′) and 805R (5′- GACTACHVGGGTATCTAATCC-3′). PCR products were confirmed via 2% agarose gel electrophoresis, purified using AMPure XT beads (Beckman Coulter Genomics, Danvers, MA, USA), and quantified using Qubit (Invitrogen, CA, USA). Amplicon pools were prepared for sequencing, and the size and quantity of the amplicon library were assessed using an Agilent 2100 Bioanalyzer (Agilent, USA) and the Library Quantification Kit for Illumina (Kapa Biosciences, MA, USA), respectively. Libraries were sequenced on the NovaSeq PE250 platform. Paired-end reads were merged using FLASH, filtered, and replicated to obtain the feature table and feature sequence. Alpha and beta diversity were calculated by normalizing to the same sequences randomly. Feature abundance was normalized using the SILVA database (release 138) based on relative sample abundance. Sequence alignment was performed using BLAST, and feature sequences were annotated using the SILVA database for each representative sequence.

### 2.9 Statistical analysis

The statistical analysis was conducted using SPSS software (version 16.0; SPSS Inc., Chicago, IL, USA). Prior to conducting a one-way analysis of variance (ANOVA), the Shapiro–Wilk test for normality and Levene’s test for homogeneity of variances were applied to each dataset group. If the data adhered to a normal distribution and exhibited homogeneity of variance, standard ANOVA procedures were employed. In instances where ANOVA revealed statistically significant differences among groups, Tukey’s Honestly Significant Difference (HSD) test was subsequently utilized for *post hoc* multiple comparisons. *p* < 0.05 indicated that the differences were statistically significant.

## 3 Results

### 3.1 Analysis of major components in TCBR

**Figure 1:**
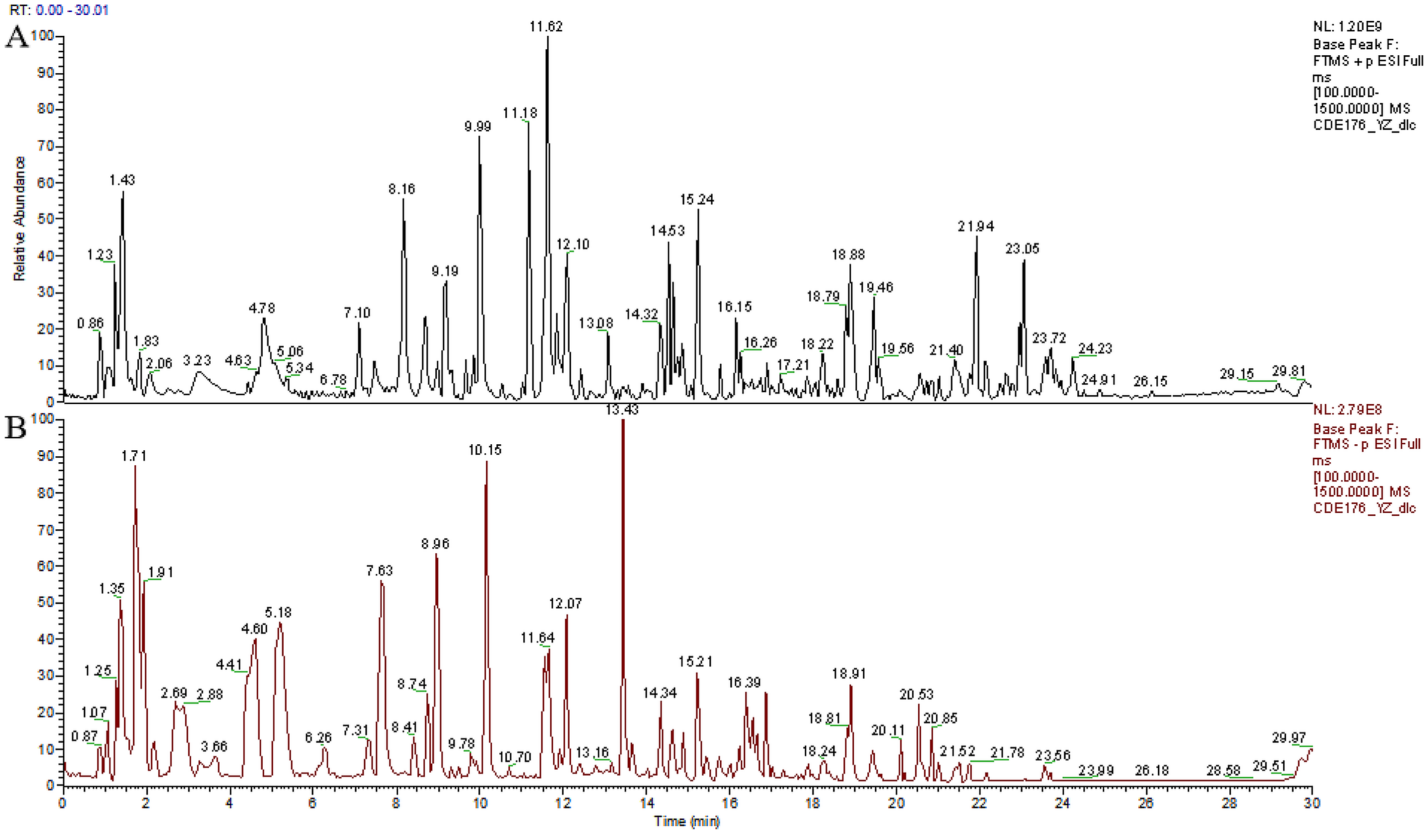
Base peak intensity chromatograms of TCBR in ESI+ **(A)** and ESI− **(B)** modes.

To evaluate the potential of TCBR as a functional feed, our study employed the UPLC technique to identify the constituents of TCBR (Figures 1A,B). The results revealed that the top10 primary active ingredients in TCBR are Vitexin, Naltrexone, Citric Acid, Ethylmorphine, Azelaic Acid, Phenibut, Kuromanin, Esculetin, and Ethamivan (Table 3).

**Table 3:** Major components of in TCBR.

Name	Formula	m/z	RT [min]	mzCloud Best Match	Area
Vitexin	C_21_H_20_O_10_	433.11227	12.079	96.6	3,483,825,614
Naltrexone	C_20_H_23_NO_4_	342.1698	8.681	62.8	2,785,301,371
Citric acid	C_6_H_8_O_7_	191.01915	2.699	97.7	1,648,858,701
Ethylmorphine	C_19_H_23_NO_3_	314.17465	7.098	73	1,624,209,722
Azelaic acid	C_9_H_16_O_4_	187.09666	13.438	97.3	1,174,633,063
Phenibut	C_10_H_13_NO_2_	180.10185	1.432	79.4	841,702,243
Kuromanin	C_21_H_20_O_11_	449.10748	13.554	37.1	405,646,610
Esculetin	C_9_H_6_O_4_	179.03398	10.149	97.9	149,301,487
Ethamivan	C_12_H_17_NO_3_	224.12817	8.961	42.7	89,835,224
Propamocarb	C_9_H_20_N_2_O_2_	188.15248	1.084	35.6	71,533,316

### 3.2 Clinical records, growth performance and slaughter performance

**Figure 2:**
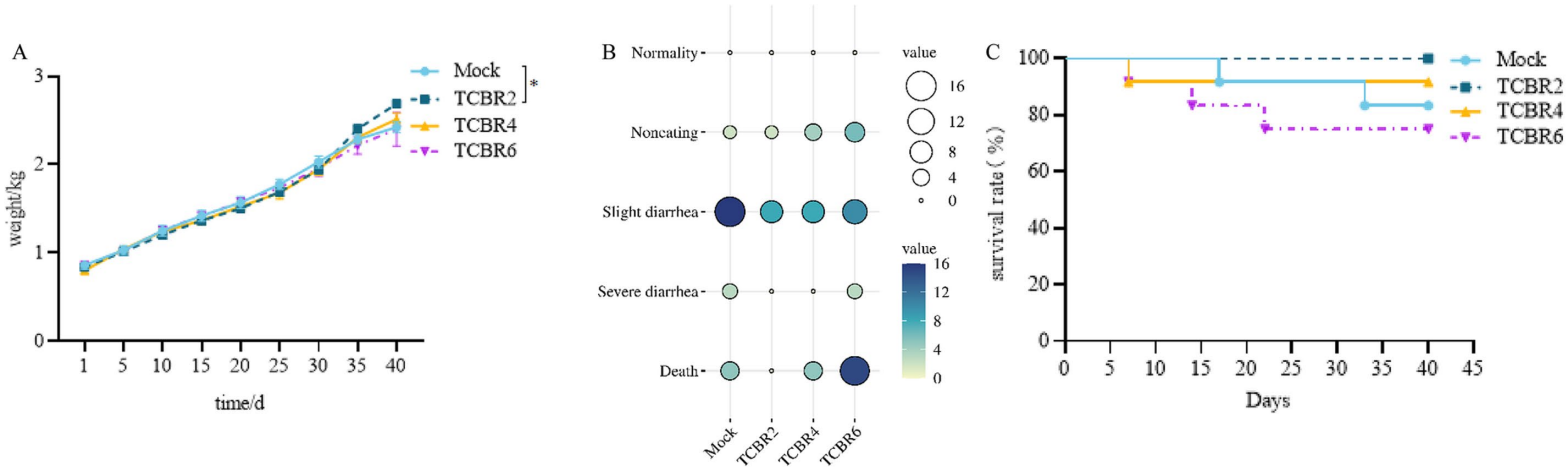
Clinical condition documentation for rabbits. **(A)** Recorded body weights of rabbits. Values represent means of eight replicate samples. *p < 0.05 compared to the Mock group. **(B)** Clinical performance score statistics: scores range from 0 to 5, with higher scores indicating more severe disease; darker colors represent more disease cases. **(C)** Survival rates of rabbits.

To investigate whether TCBR showed a positive effect on weaned rabbits, we recorded clinical symptoms, growth performance and slaughter performance of weaned rabbits. TCBR2 significantly increased the BW of rabbits (13.4%) compared with those in the Mock group (*p* < 0.05). However, the TCBR4 and TCBR6 groups did not show significant differences (Figure 2A). TCBR2 also improved the rabbit survival rate and reduced their adverse clinical manifestations (diarrhea and death; Figures 2B,C). The Mock group had the highest diarrhea index (Figure 2B), whereas the TCBR6 group had the highest mortality rate (33.3%) (Figure 2C).

Compared with that in the Mock group, TCBR2 increased the BW, average daily BW gain, and FCR of rabbits between 37 and 72 days of age (*p* < 0.05; Table 4). However, TCBR4 and TCBR6 showed no notable benefits. TCBR2 significantly increased carcass yield and half-eviscerated carcass yield by 3.1 and 4.1%, respectively, compared with the Mock group (*p* < 0.05; Table 4). No significant differences were observed in the carcass yield and half-eviscerated carcass yield between the TCBR4 and TCBR6 groups (*p* > 0.05).

**Table 4:** Effects of Trollius chinensis Bunge residue supplementation on the growth and slaughter performance of rabbits^1^.

Item	Mock^2^	TCBR2	TCBR4	TCBR6	SEM	*p*-value
Initial BW, g	850	837.5	779.2	875	0.02	0.19
BW, g	2371.4^a^	2688.9^b^	2514.3	2250.0	0.06	0.01
Average daily gain, g	42.0^a^	52.9^b^	49.6	39.6	1.89	0.01
Average daily feed intake, g	151.8	147.9	145.3	156.7	2.58	0.43
Feed conversion ratio	3.7^a^	2.7^b^	3.0	3.8	0.18	0.02
Carcass yield, %	81.8^a^	84.9^b^	83.0	81.7	0.44	0.01
Partial-eviscerated carcass yield, %	56.4^a^	60.5^b^	59.6	58.3	0.70	0.03
Eviscerated carcass yield, %	47.0	49.3	48.8	47.8	0.67	0.67

1 Values are means of eight replicate samples.

2 The mock group was fed a basal diet; TCBR2, TCBR4, and TCBR6 indicate that 2, 4, and 6% of the Trollius chinensis Bunge residue supplementation on the control diet, respectively.

^a,b^Different superscript letters between treatments indicate significant differences at *p* < 0.05.

### 3.3 Serum biochemistry

To assess the toxicity of TCBR, rabbit serum was analyzed for biochemical indices. Different levels of TCBR supplementation reduced the serum glucose levels in 72-day-old rabbits (Table 5). TCBR2 and TCBR4 lowered the activities of ALP and ALT, as well as the blood urea nitrogen concentration in serum, compared with the Mock treatment. TCBR2 also increased the levels of total protein compared with the Mock treatment. Additionally, TCBR6 significantly increased serum total cholesterol concentration (*p* < 0.05).

**Table 5:** Effects of Trollius chinensis Bunge residues on the serum biochemical indexes of rabbits^1^.

Item	Mock^2^	TCBR2	TCBR4	TCBR6	SEM	*p*-value
GLU, mmol/L	10.2^a^	6.2^b^	7.1^b^	7.4^b^	0.44	<0.001
TG, mmol/L	1.1	0.4	0.8	1.8	0.18	0.015
T-CHO, mmol/L	0.7^a^	1.0	1.1	2.1^b^	0.16	0.001
ALB, g/L	15.5	19.2	18.0	18.3	0.63	0.197
ALT, U/L	24.0^a^	11.3^b^	13.8^b^	26.0	1.95	<0.001
BUN, mmol/L	9.2^a^	5.6^b^	5.7^b^	10.5	0.63	<0.001
ALP, U/ml	16.2^a^	9.9^b^	11.1^b^	20.8	1.42	0.007
TP, g/L	21.2^a^	22.1^b^	20.0	20.4	0.24	<0.001

1 Values are means of eight replicate samples.

2 The mock group was fed a basal diet; TCBR2, TCBR4, and TCBR6 indicate that 2, 4, and 6% of the Trollius chinensis Bunge residue supplementation on the control diet, respectively.

^a,b^Different superscript letters between treatments indicate significant differences at *p* < 0.05.

### 3.4 Antioxidant capacity

As the intervention of low-dose TCBR improved the clinical performance of rabbits, we hypothesized that TCBR may have antioxidant effects. TCBR4 and TCBR6 significantly increased CAT activity in serum (*p* < 0.05; Table 6). However, no significant differences were observed in the serum activities of SOD, GSH-Px, and MDA among treatments. In the liver, different levels of TCBR supplementation increased SOD activity, with TCBR6 also enhancing GSH-Px activity. But TCBR2 and TCBR4 decreased the concentration of MDA in the liver. However, TCBR supplementation had no effect on CAT activity in the liver.

**Table 6:** Effects of Trollius chinensis Bunge residues on the antioxidant capacity of rabbits^1^.

Item	Mock^2^	TCBR2	TCBR4	TCBR6	SEM	*p*-value
Serum						
SOD, U/ml	13.8	14.2	13.6	13.5	0.22	0.681
GSH-Px, U/ml	596.5	714.8	853.8	519.7	56.68	0.171
CAT, U/ml	2.3^a^	1.9	4.4^b^	4.9^b^	0.40	0.002
MDA, nmol/ml	0.09	0.10	0.12	0.11	0.01	0.026
Liver						
SOD, U/mg protein	69.8^a^	389.3^b^	411.9^b^	458.3^b^	56.45	0.043
GSH-Px, U/mg protein	26.4^a^	28.0	27.2	31.4^b^	0.74	0.049
CAT, U/mg protein	2.7	3.2	3.3	2.6	0.14	0.182
MDA, nmol/mg protein	0.60^a^	0.29^b^	0.27^b^	0.64	0.46	<0.001

1 Values are means of eight replicate samples.

2 The mock group was fed a basal diet; TCBR2, TCBR4, and TCBR6 indicate that 2, 4, and 6% of the Trollius chinensis Bunge residue supplementation on the control diet, respectively.

^a,b^Different superscript letters between treatments indicate significant differences at *p* < 0.05.

### 3.5 Jejunal morphology

**Figure 3:**
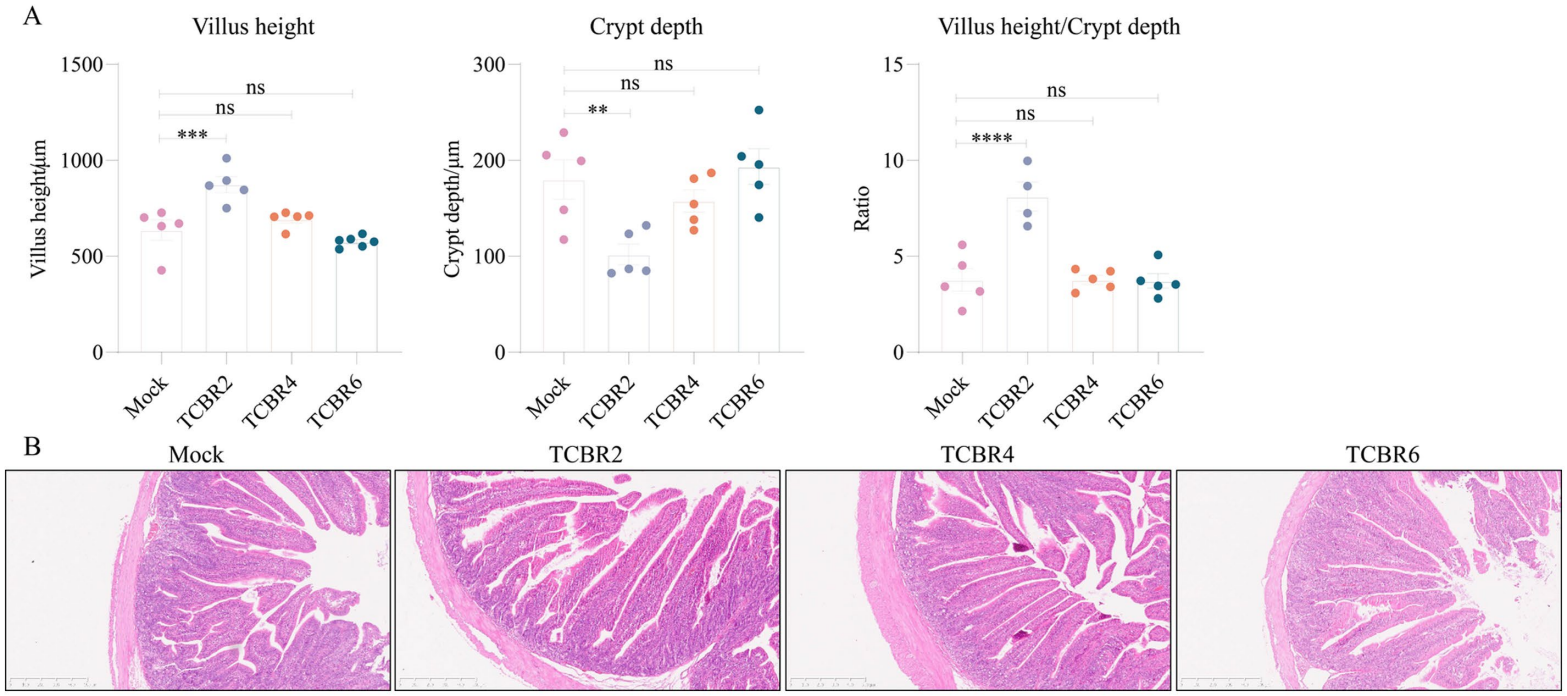
Effects of Trollius chinensis Bunge residue supplementation on the jejunal morphology of rabbits. **(A)** Villus height, crypt depth, and the villus-to-crypt ratio among treatments. Values represent means of eight replicate samples. *p < 0.05, **p < 0.01, ***p < 0.001, and ****p < 0.0001 compared to the Mock group. **(B)** Representative images of hematoxylin and eosin–stained jejunal tissue. Scale bar: 500 μm.

In addition, supplementation of low-dose TCBR increased body weight in rabbits, and we speculated that perhaps TCBR showed a positive effect on rabbits intestinal tissues. The jejunal morphology of tested rabbit is shown in Figure 3. Compared with Mock rabbits, those supplemented with TCBR2 showed significantly increased villus height and V/C ratio (*p* < 0.001; Figure 3A). However, no significant differences were found in villus height or the V/C ratio with TCBR4 and TCBR6 supplementation (*p* > 0.05) compared with the Mock treatment. Jejunal sections, stained with hematoxylin and eosin, are shown in Figure 3B.

### 3.6 Gene expression

**Figure 4:**
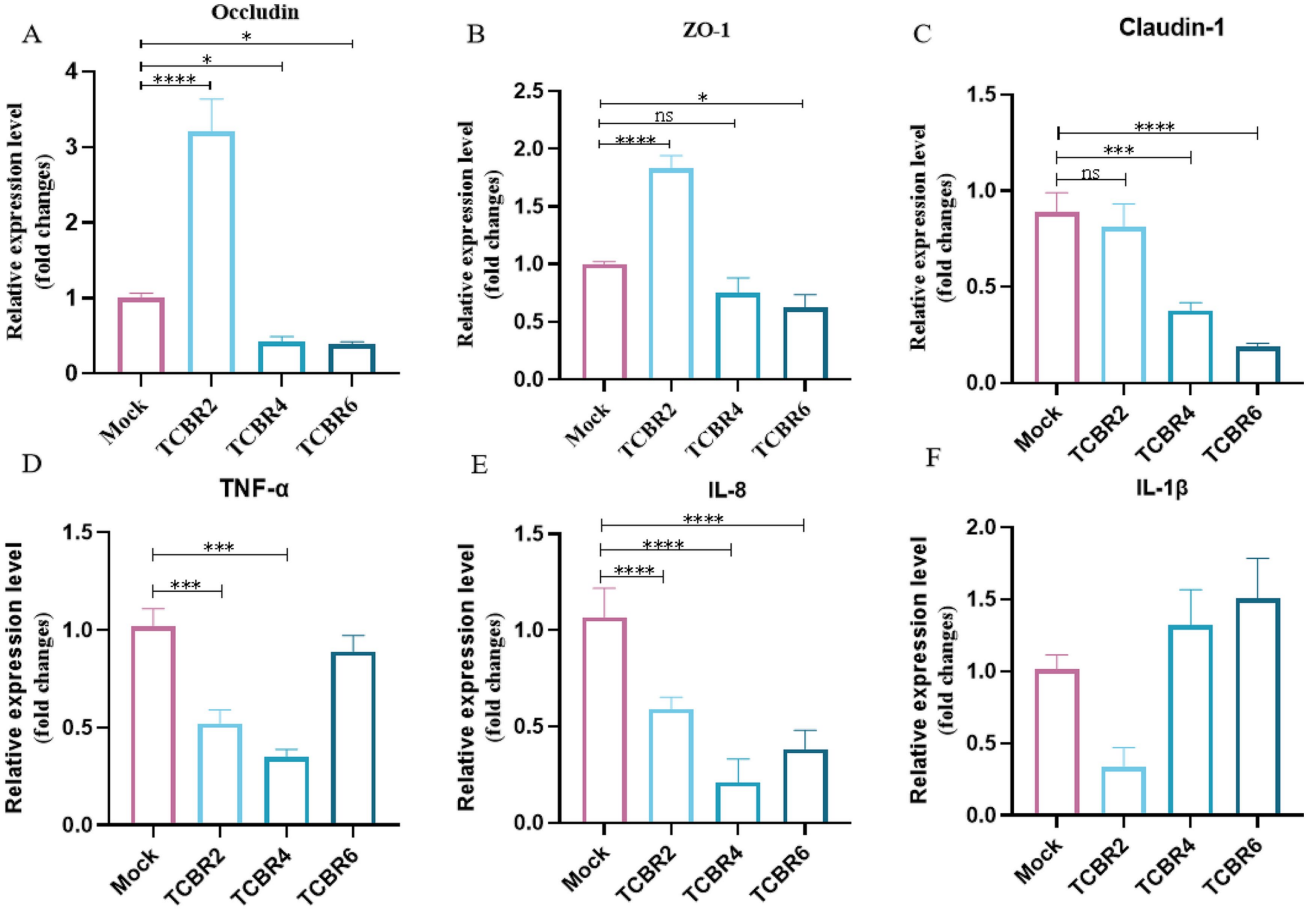
Relative mRNA expression levels of genes in the jejunal mucosa of rabbits. **(A–C)** Show jejunal mechanical barrier gene expression levels. **(D–F)** Indicate jejunal inflammatory gene expression levels. *p < 0.05, **p < 0.01, ***p < 0.001, and ****p < 0.0001 compared with the Mock group.

Meanwhile, we also evaluated inflammatory factors and gut barrier in the gut. The effects of TCBR supplementation on gene transcript levels in the jejunal tissue of rabbits are shown in Figure 4. Compared with the Mock treatment, TCBR2 supplementation significantly up-regulated the expression levels of occludin and ZO-1 but had no significant effect on claudin-1 expression. In contrast, TCBR4 and TCBR6 supplementation down-regulated the expression levels of occludin, ZO-1, and claudin-1 in the jejunal tissue. Additionally, TCBR2 and TCBR4 supplementation significantly down-regulated the relative expression levels of TNF-*α* and IL-8 (*p* < 0.05).

### 3.7 Cecal microbiota analysis

**Figure 5:**
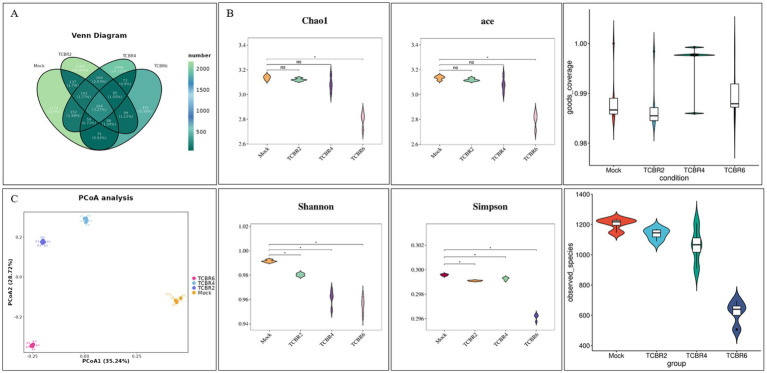
Cecal microbiota richness and diversity. **(A)** ASV Venn diagram. **(B)** Comparison of alpha diversity indexes presented as box plots. **(C)** *β*-diversity analysis. *n* = 5, *Significant difference at *p* < 0.05.

In our study, 8,081 ASV were obtained through sequencing, with the number of operational taxonomic units decreasing as TCBR concentration increased (Figure 5). ASV Venn analysis identified 2,174, 2,169, 1948, and 451 unique ASV in the Mock, TCBR2, TCBR4, and TCBR6 groups, respectively (Figure 5A). Compared with the Mock group, both the ACE and Chao1 indexes were reduced in the TCBR6 groups (*p* < 0.05). The Shannon and Simpson indexes were lower in all TCBR-treated groups relative to the Mock group (*p* < 0.05). Moreover, the goods_coverage index is above 0.98 for all samples. That indicated high coverage of species in the samples and reasonable sequencing depths. Principal coordinate analysis indicated a progressive shift in the microbial community with increasing dietary TCBR levels (Figure 5C).

**Figure 6:**
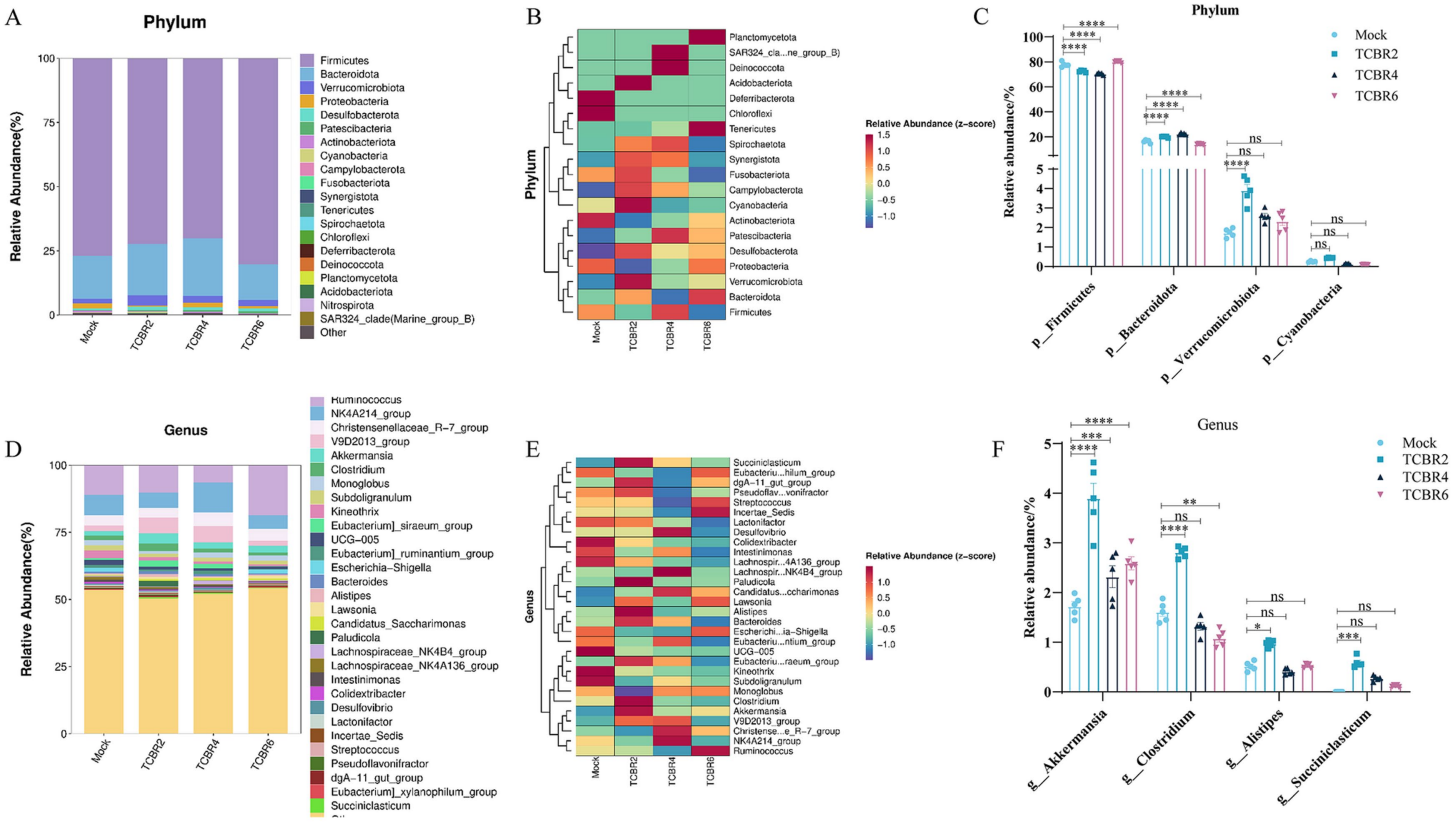
Relative abundance and differences in cecal microbiota. **(A)** Relative abundance of the top 20 phyla. **(B)** Heatmap of the top 20 phyla. **(C)** Differences in microbiota composition at the phylum levels. **(D)** Relative abundance of the top 30 genera. **(E)** Heatmap of the top 30 genera. **(F)** Differences in microbiota composition at the genus levels. *p < 0.05, **p < 0.01, ***p < 0.001, and ****p < 0.0001 compared to the Mock group.

TCBR2 and TCBR4 supplementation reduced the relative abundance of the cecal Firmicutes phylum while increasing the relative abundance of the cecal *Bacteroidota* phylum (*p* < 0.0001; Figure 6). TCBR2 also increased the relative abundance of the cecal *Verrucomicrobiota* phylum (*p* < 0.0001). All TCBR levels increased the relative abundance of the cecal *Akkermansia* genus (*p* < 0.001). TCBR2 further increased the relative abundance of the cecal *Clostridium*, *Alistipes*, and *Succiniclasticum* genera (*p* < 0.05), whereas TCBR4 reduced the relative abundance of the cecal *Clostridium* genus (*p* < 0.05).

**Figure 7:**
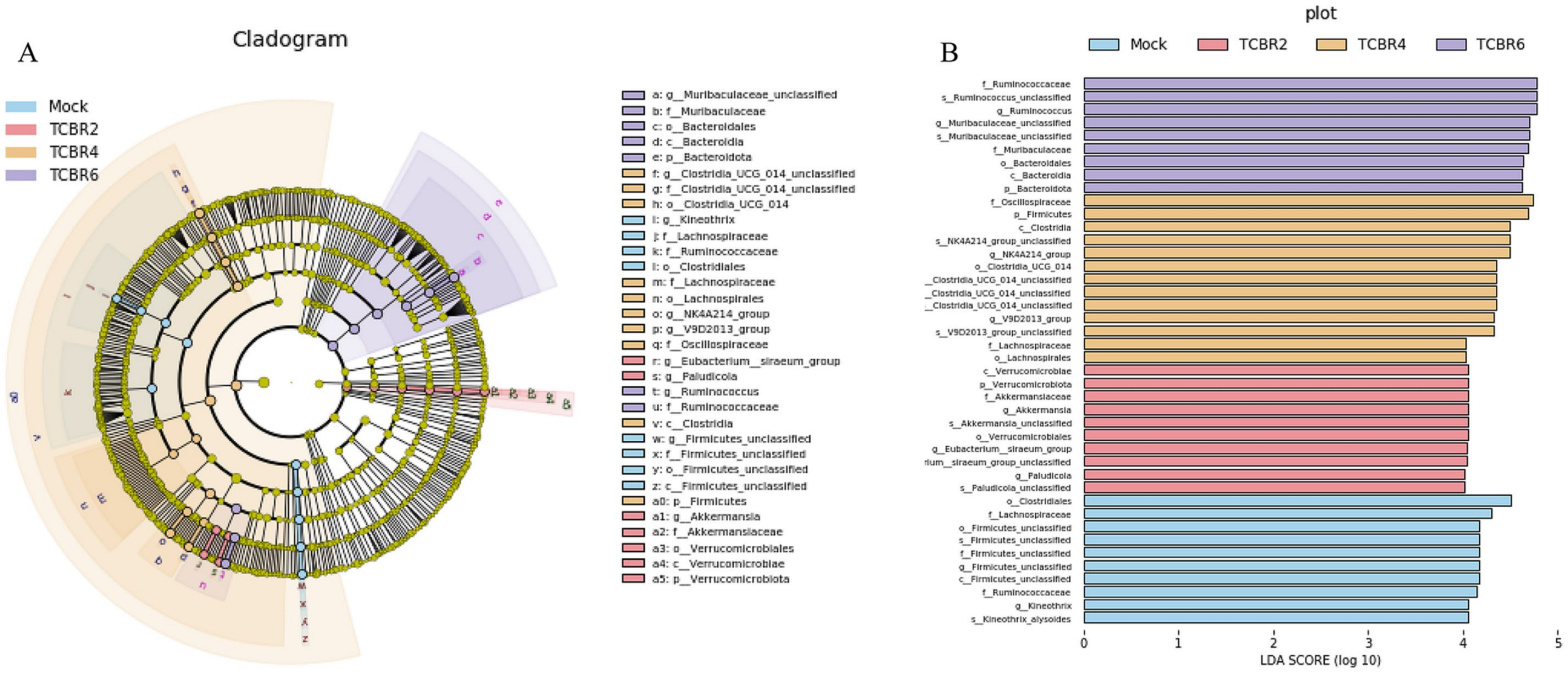
Linear discriminant analysis (LDA) effect size (LEfSe) in cecal microbiota. **(A)** Evolutionary branching diagram. Circles radiating from inside to outside represent taxonomic levels from phylum to genus (or species). Each small circle at a different taxonomic level represents a taxon at that level, and the size of the diameter of the circle is proportional to the relative abundance size. **(B)** LDA value distribution histogram. The different colored zones represent different groups, with the four colors indicating microbial taxa that are significant in the four groups, respectively. Only species with an LDA Score > 4 are displayed in the diagram, and the length of the bars represents the magnitude of the LDA values. Method: Kruskal-Wallis rank sum test.

We conducted LefSe analysis to elucidate the differences in microbial abundance between the Mock and TCBR-treated groups. As exhibited in Figures 7A,B, a total of 42 branching taxa showed significant differences in abundance (LDA Score > 4; *p* < 0.05). Among these, 10 branch taxa were evaluated to be significantly affected in the Mock group, including *o_clostridiales*, *f_lachnospiraceae*, *o_firmicutes*, and others. Subsequently, the TCBR2 group showed considerable consequences for 10 branch taxa, comprising *c_verrucomicrobiae*, *f_akkermansiaceae*, *g_paludicola*, and others. But 13 branch taxa were exhibited to be significantly affected in the TCBR4 group, containing *f_oscillospiraceae*, *p_firmicutes*, *c_clostridia*, and others. In addition, TCBR6 group demonstrated substantial influence on 9 branch taxa, consisting of *f_ruminococcaceae, g_muribaculaceae*, *o_bacteroidales*, and others.

## 4 Discussion

Rabbits are considered ideal meat-producing animals owing to their short life cycle, short gestation period, high reproductive rates, and efficient feed conversion. As an important source of meat protein, the rabbit farming industry is receiving increasing attention (1). However, gut health of weaned rabbits is important for rabbit growth, with the growing emphasis on reducing antibiotic use in the livestock industry, there are higher demands for the sustainable and healthy development of rabbit farming. Previous study suggested that *Taxus chinensis Rehder* fruit extract (TCFE) demonstrated significant anti-aging properties by reducing microglia activation, decreasing oxidative stress, and influencing inflammatory pathways (33). meanwhile, *Canna x generalis* extract (CGE) improved the integrity of the intestinal mucosal barrier and lessened oxidative stress and inflammation (34, 35). Which indicated that Traditional Chinese medicine plays a role in promoting animal health and improving production efficiency. Their byproducts, residues left after the extraction of active herbal ingredients, may still contain active compounds beneficial to livestock (9, 36, 37). However. The specific role of TCBR as a feed additive for weaned rabbits remains to be elucidated. A previous study showed that adding turmeric residue improved survival rates in Chinese soft-shelled turtles exposed to high ambient temperatures (38). He et al. found that supplementing Shengxuebao herbal residues in the diets of heat-stressed New Zealand rabbits increased BW (39). Similarly, our study found that adding 2% TCBR significantly increased final BW, reduced adverse clinical symptoms, and improved survival rates in weaned rabbits. TCBR2 also significantly increased the average daily BW gain of the rabbits. These effects may be linked to the presence of bioactive substances, such as brassinoids, organic acids, and alkaloids, in TCBR. However, high dose of Trollius chinensis Bunge residues, with 6% in the rabbits diet, showed adverse clinical syndrome, suggesting potential toxicity at highier dose. The study found that flavonoids produced cellular damage at 450 μM cellular viability, causing a 12–60% reduction in cellular viability in isolated guinea pig enterocytes and lactic dehydrogenase (LDH) leakage 28–41% greater (40). Moreover, the total flavonoids (TFs) from *Rosa laevigata* Michx fruit could cause side effects at the dose of 2000 mg/kg/day in males and females, such as increased intercellular space of myocardial cells and the relative cardiac weight (41). In addition, Aconitine, a component of traditional Chinese medicine, showed to induce cardiotoxicity and neurotoxicity at high doses, resulting the destruction of neuronal cells (42). Concurrently, Excessive exposure to Tripterygium hypoglaucum and *Myrica rubra* pomace polyphenols (MRPP) has been linked to damage in hepatocytes and the intestines (43, 44). meanwhile, pyrrolizidine alkaloids (PAs) derived from Eupatorium fortunei demonstrated to cause significant hepatotoxicity by disrupting glycerophospholipid metabolism at a dosage of 25 mg/(kg-day) in mouse model (45). Therefore, Our study provided an experimental basis for the use of TCBR as a feed additive, and its practical application needs to be further verified by a larger-scale sample.

Slaughter rate is a key indicator of growth and performance. A previous study found that supplementation with TCM significantly increased the slaughter and evisceration rates of broiler chickens (46). Similarly, supplementation on *Eucommia ulmoides* polysaccharides to diets improved growth and carcass performance in Songliao black pigs (47). Moreover, dietary supplementation of *Litsea cubeba* essential oil (LCO) and *Alpinia oxyphylla* essential oil (AEO) to fattening pigs improves growth performance and the efficiency of digestion and absorption of nutrients (48, 49). In our study, TCBR2 supplementation significantly increased the carcass and partial-eviscerated carcass yield in rabbits, consistent with findings from previous studies.

Blood biochemical markers, such as sugars, fats, and proteins, provide insight into an animal’s growth, nutrient digestion, absorption, and metabolism. For instance, albumin, total protein, and urea nitrogen levels reflect protein and amino acid utilization and metabolism, whereas blood glucose levels indicate an animal’s glucose regulation and overall health, and triglyceride and total cholesterol levels suggest the efficiency of fat metabolism (50, 51). Our study revealed that TCBR2 supplementation significantly increased serum total protein levels while decreasing urea nitrogen and triglyceride levels. Moreover, TCBR supplementation lowered serum glucose levels. And the Chinese medicine *Tongxinluo* significantly reduced lipid levels in rabbits (52). However, Some studies indicated that *Ligustrum lucidum* supplementation does not significantly impact glucose, total protein, or albumin levels in Hy-Line Brown hens during the late laying period (53), which may be attributed to differences in the animal species and herbs.

The host metabolism is regulated by the gut microbiome. Short-chain fatty acids, branched-chain amino acids and bile acids synthesized by the gut microbiome are a direct source of energy for the host cells and also affect the energy metabolism of its host (54). Previous study showed that *Portulaca oleracea* increased the metabolic tryptophan and some vitamins in the intestinal flora, thereby promoting intestinal health and growth performance in Hu Lambs (55). Wei et al. also demonstrated that supplementation of dairy cows with low crude protein (CP) and rumen-protected lysine (RPL) balanced the amino acid supply, increased the efficiency of nitrogen utilization, and altered the intestinal flora composition, which was beneficial to the lactation performance of the cows (56). ALT and ALP are cytokines of liver metabolism and important indicators of liver injury (57). Researcher suggested that *Gegen-Qinlian* decoction (GQD) alleviated liver injury and metabolic disorders in Metabolic dysfunction-associated steatohepatitis (MASH) mice by correcting intestinal dysbiosis and modulating the bile acid (BA) profile in a mouse model of metabolic dysfunction-associated steatohepatitis (58). TCBR2 supplementation in rabbits reduced serum ALT and ALP levels but significantly increased the liver SOD activity; however, it had no significant effect on GSH-Px and catalase levels. A previous study showed that supplementing with *Ilicis Chinensis folium* extract significantly reduced ALT levels and increased SOD activity in broiler chickens (59). Similarly, acidic polysaccharides from Schisandra chinensis significantly reduced ALT levels and increased SOD activity in cases of liver injury (60). Additionally, researcher verified that hydroxysafflor yellow A (HSYA) treated traumatic brain injury (TBI) mice mainly through affecting the functions of blood vessels, increased brain microvessel density and the expression of angiogenic marker proteins VEGFA and CD34 (61). Moreover, *Qiwei Tiexie* pills (QWTX) effectively attenuates acetaminophen (APAP) overdose-induced elevation of serum AST, ALT and inflammatory factors in a mouse model (62). Furthermore, supplementation with 4 and 6% TCBR significantly increased serum CAT levels. These results indicated that TCBR maybe possess antioxidant properties.

As a crucial organ for nutrient absorption, the intestines play a key role in maintaining overall health through their structure, mucosal barrier, and microbial composition. We hypothesize that components within TCBR can regulate intestinal flora and modify the intestinal index. Previous research has shown that various compounds, such as patchouli alcohol, luteolin, and other therapeutic agents, can regulate gut flora and reduce intestinal inflammation and damage (63–65). Our findings suggested that TCBR2 supplementation significantly increased jejunal villus height and decreased crypt depth compared with the Mock treatment. The intestinal barrier, consisting of microbial, mechanical, and chemical components, plays a key role in preventing harmful substances from entering the bloodstream and maintaining the body’s internal stability (66). TCBR2 supplementation in our study significantly up-regulated the relative expression of occludin-1 and ZO-1 while down-regulating the relative expression of TNF-*α* and IL-8. The tight junction proteins occludin and ZO-1 are vital for maintaining the integrity of the intestinal mucosa. Modified *Gegen Qinlian* decoction has been shown to improve symptoms and pathological damage in ulcerative colitis model mice by up-regulating occludin and ZO-1 expression (67). Mulberry extract was found to attenuate colonic damage and inflammation, suppress colonic oxidative stress, and restore intestinal tight junction protein expression in mice with DSS-induced colitis (18). Our research showed that TCBR2 supplementation significantly increased the relative abundance of the Bacteroidetes and *Verrucomicrobiota* phylum, particularly the *Akkermansia* genus. *Bacteroidetes*, a prominent bacterial group within the animal gut microbiome, significantly influence host growth by facilitating food digestion and nutrient absorption (68). These bacteria function as intestinal symbionts by providing protection against pathogens and supporting other gut microorganisms (69). Researcher demonstrated that mouse pretreated with *Mycobacterium anthropophilum* spp. and its metabolites enhanced survival rates, reducing brainstem inflammation after infection HSV-1 (70). Additionally, *Akkermansia*, a genus of anaerobic Gram-negative bacteria from the Verrucomicrobiota phylum, resides in the gut lining and plays a crucial role in maintaining gut health (71). The presence of *Akkermansia muciniphila* is strongly associated with positive effects on host metabolism, efficacy of cancer treatment checkpoints, and the maintenance of immune stability. Research demonstrated that TLR2-TLR1 heterodimers play a regulatory role in modulating lipid diacylphosphatidylethanolamine within the cell membranes of *Akkermansia*, thereby promoting a preference for TNF-*α* production (72). In a murine sepsis model, the administration of *Akkermansia* significantly reduced sepsis-induced mortality. The Arg-Lys-His (RKH) protein secreted by *Akkermansia* binds directly to Toll-like receptor 4 (TLR4), inhibiting TLR4 signaling in immune cells. This interaction results in decreased activation of inflammatory cells and a reduction in the overproduction of pro-inflammatory factors associated with sepsis (73). Similarly, The gut flora can secrete SCFA, potent anti-inflammatory agents, inhibited neutrophils and macrophages from releasing pro-inflammatory cytokines, to maintain epithelial integrity, thereby contributing to immune system stability. Previous study demonstrated that butyrate enhances the expression of the tight-junction proteins claudin-1 and ZO-1 and upregulate the expression of mucin 2 (MUC2) to reinforce the mucous layer against luminal pathogens (74). Through multi-omics sequencing and correlation analysis, it was found that butyric acid (BA) produced by *Faecalibacterium prausnitzii* plays a crucial role in improving valve function in calcific aortic valve disease (CAVD) (75). Interestingly, TCM is not only effective in disease through the gut-liver axis, but can also positively affect disease through the gut-brain axis. Yu et al. demonstrated the significant regulatory impact of *Ginkgo biloba* extract on intestinal microflora and microbial metabolism in Alzheimer’s disease (AD) model mice, reducing the *Firmicutes/Bacteroides* ratio and increasing *Bacteroidetes* abundance (76). and *Lonicerae Japonicae* Flos (LJF) may have a therapeutic effect in AD (77). Importantly, *Astragaloside IV* likely prevents the proliferation and migration of microglia/macrophages after intracerebral hemorrhage (ICH) by binding its transformed products to CDC42, PTK2, and CSF1R in C57 BL/6 mice model (78). Moreover, ASIV altered the gut microbiota, and inhibited the production of conditional pathogenic bacteria. Li et al. suggested that gut microbiota and its metabolites may be the key regulator of AS-IV in treating ICH (79). In brief, TCM showed promising prospects in the treatment of various diseases and multiple applications.

## 5 Conclusion

**Figure 8:**
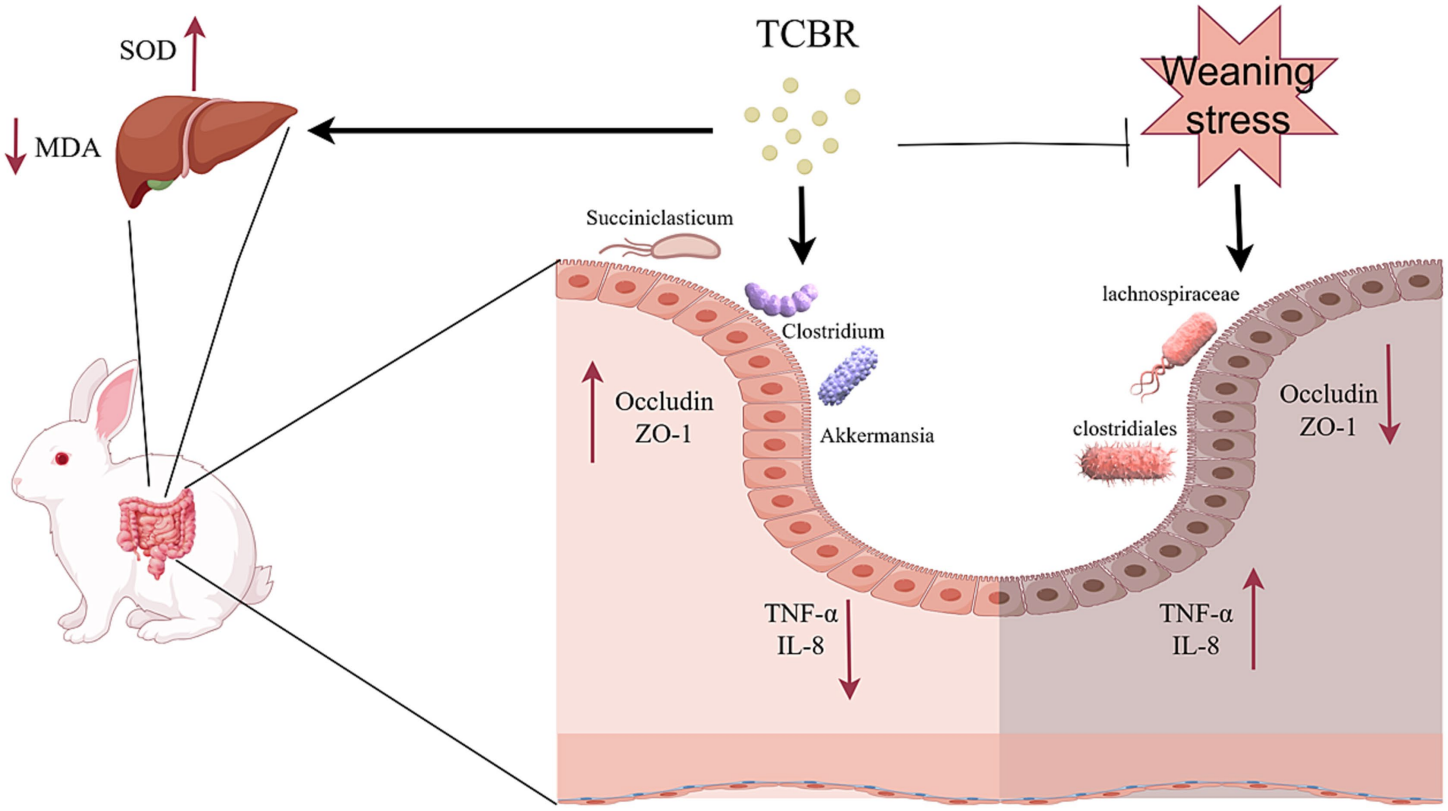
Prediction of the pathways involved in TCBR’s actions.

The inclusion of 2% TCBR in the diets of growing rabbits alleviated adverse clinical symptoms and improved survival rates, growth performance, and meat quality by increasing BW and BW gain while decreasing the FCR. These benefits may be attributed to enhanced antioxidant activity in the liver, improved jejunal morphology, and a higher relative abundance of beneficial intestinal flora (Figure 8). These findings highlight the potential of TCBR as an effective feed additive in rabbit production.

## Data Availability

The data presented in the study are deposited in the NCBI repository, accession number BioProject: PRJNA1322195.
